# Characterization of pathogenic *Enterococcus cecorum* from different poultry groups: Broiler chickens, layers, turkeys, and waterfowl

**DOI:** 10.1371/journal.pone.0185199

**Published:** 2017-09-21

**Authors:** Beata Dolka, Dorota Chrobak-Chmiel, Michał Czopowicz, Piotr Szeleszczuk

**Affiliations:** 1 Department of Pathology and Veterinary Diagnostics, Faculty of Veterinary Medicine, Warsaw University of Life Sciences-SGGW, Warsaw, Poland; 2 Department of Preclinical Sciences, Faculty of Veterinary Medicine, Warsaw University of Life Sciences-SGGW, Warsaw, Poland; 3 Laboratory of Veterinary Epidemiology and Economics, Faculty of Veterinary Medicine, Warsaw University of Life Sciences-SGGW, Warsaw, Poland; University of Campinas, BRAZIL

## Abstract

*Enterococcus cecorum* (EC) is known as a commensal in the intestines of mammals and birds. However, it has been described as an emerging pathogen in poultry industry worldwide. The aim of this study was to analyze and compare EC isolated from clinical material collected from poultry groups with different production purposes. The genetic diversity among pathogenic EC in relation to each specific poultry type was examined. In total, 148 isolates from independent infection outbreaks (2011–2016) were used: 76 broiler chickens (CB), 37 broiler breeders (BB), 23 layers (CL), 7 waterfowl (W) and 5 turkey (T) flocks (1 isolate/1 flock). We provided age ranges at diagnosis of EC-infection for 5 poultry groups. Isolates obtained from CB were significantly more frequently retrieved from bone marrow, joints, spine, and contrary to BB, CL less frequently retrieved from respiratory system. The study showed differences between EC of various poultry types in relation to 10/32 (31.3%) biochemical parameters. EC isolates from CB were significantly more often positive for βGAL, βNAG, MLZ, and less often positive for PAL and βMAN than isolates from other poultry types. However, BB and W isolates showed higher ability to metabolise mannitol than CB, CL, and T. CB isolates showed lower ability to survive at 60°C. Only chicken EC-isolates harbored virulence genes: CB (8.1%) > BB (3.4%) > CL (2%). No specific pulsotype of EC was associated with a specific poultry. One or several various (up to 6) genetic types of EC may be involved in outbreaks in CB flocks within one year in one region. Outbreaks reported in following years in the same region were usually caused by a distinct set of EC-genetic types. PFGE results indicated at the genetic heterogeneity among pathogenic isolates involved in outbreaks in relation to each poultry type. To our best knowledge, this is the first study which provides a comparison between clinical EC from 5 poultry groups. The study provides a new insight into EC as pathogen of different bird species. The obtained data may be useful in further studies on EC-infections more focused on a specific type of poultry.

## Introduction

*Enterococcus cecorum* (EC) belongs to a group of Gram-positive, facultatively anaerobic, catalase- and oxidase-negative cocci. The colonies on blood agar plates are surrounded by α-haemolysis. Contrary to other enterococci this species is not assigned to the serological group D. The first isolation was described in 1983 from the caeca of healthy chickens [1]. EC is a component of normal enterococcal microbiota in the gastrointestinal tract of various birds (poultry, pigeons, canaries) and domestic vertebrates (cattle, horses, pigs, cats, dogs) [2–6]. *Enterococcus* spp. were the most often isolated bacteria from18-20-day embryos and newly hatched chickens [7]. However, EC has not been found in gut flora of 1 day-old chicks [2]. Some studies have shown age-dependent intestinal colonization by EC in chickens [8, 9]. The earliest appearance of EC in the crop, small intestine and caeca was observed at the age of 3–4 weeks. EC was the most frequently occurring commensal enterococcus in layers and parent flock of over 12 weeks of age [2].

Over the last 15 years, EC formerly viewed as a bacterium of minimal clinical impact has emerged as an important poultry pathogen. The first infection due to EC was diagnosed in 2002 in 4–5 week-old broilers in Scotland [10] and in 3 week-old broilers in the Netherlands [11]. In the following years, several outbreaks were reported in broiler chickens in Belgium, Canada, USA, Poland, Germany, Malaysia [12–18], in broiler breeders in USA, Canada, Hungary, South Africa, Iran [13, 14, 19–24], in meat turkeys in Canada [23] and in ducks in Germany [24].

EC has a predilection for cartilages and bones, especially for free thoracic vertebra—Th6 (FTV) in young broiler males [21]. Infection due to EC may result in vertebral abscess formation causing spinal cord compression and clinical signs such as lameness and arching of the back, birds sitting down on hocks, paralysis and deaths. Recent studies revealed differences between commensal and pathogenic EC strains isolated from different animals [25]. Our previous study was focused on the phenotypic and genotypic characterization of poultry clinical EC isolates treated as a one whole group [26]. Despite recent advances in the understanding of this bacterium and pathogenesis of EC infection [27, 28], little is known about the properties of pathogenic strains responsible for diseases in various poultry species with different production purposes. Additionally, little information is available on EC infection in laying hens or geese. In the view of the above, here we have focused on specific poultry types and performed comparative analysis of pathogenic *E*. *cecorum* from five different poultry groups (commercial chickens broilers, broiler breeder chickens, commercial chickens layers, turkeys, waterfowl). In contrast to our previous study, here the poultry groups were treated separately.

This study aimed to find and identify differences in biochemical features, ability to grow, survival, and carriage of genes encoding virulence factors between clinical EC strains isolated from five poultry groups and to analyze the genotype profiles of EC within each poultry group. Moreover, we have analyzed the age at time of EC infection diagnosis, the incidences of EC isolation from different tissue samples and pathological lesions for poultry groups.

## Materials and methods

### Flock data and bacterial isolation

The collection comprised of 148 clinical EC isolates taken between 2011–2016 and originated from 76 commercial broiler chicken flocks (CB), 37 broiler breeder flocks (BB), 23 commercial layer flocks (CL), 5 turkey flocks (T), and 7 waterfowl (W) including: 6 geese flocks (G) and 1 duck flock (D). Eighty two isolates were from the previous study. New isolates represents 80.5% more in comparison to the previous study [26]. Isolates were obtained from archival bacterial collection deposited at the Division of Avian Diseases, or were obtained from clinical specimens submitted for routine diagnostic work to the Division of Avian Diseases, Faculty of Veterinary Medicine at the Warsaw University of Life Sciences-SGGW (Poland). Samples were collected for laboratory diagnosis as a part of the usual veterinary practice, and ethical guidelines and animal welfare regulations were strictly respected. The aim of the study has determined the use only a single isolate from affected flock. The criteria used in choosing the representative isolates were: only clinical *E*. *cecorum* isolates, from different years or locations, retrieved from organs demonstrating pathological lesions. In cases of multiple isolates, the isolate from the bone marrow (typical spinal abscesses or joint lesions) was chosen at first (especially when locomotor problems were reported), then isolate from heart, and other organs (liver, spleen, respiratory duct) depending on the severity of macroscopic lesions. Two isolates (CB, BB) were retrieved from yolk sacculitis (deaths during the first week of age). Two CB isolates were retrieved from dead-in shell-embryos. One CL isolate was retrieved from ovary and one from deformed eggs. Archival isolates were not specifically collected for this study, however all fulfilled a criteria adopted in this study. Most of them were retrieved from bone marrow or heart samples. The samples were plated onto Columbia agar supplemented with 5% sheep blood (CA, Graso, Poland) and agar plates containing esculin (Enterococcosel Agar, Graso, Poland), then incubated at 37°C for 24h in a CO_2_-enriched atmosphere. As the study was focused on bacterial isolates collected from routine samples, Ethics Committee approval was not required for presented work according to the European Union regulations.

### Bacterial identification and evaluation of phenotypic properties

All isolates were previously identified to the genus based on the colony morphology, type of hemolysis, esculin hydrolysis, and catalase reaction. Then, colonies suspected to be *Enterococcus* were identified to the species level by API rapid ID 32 STREP (bioMérieux, France). The API test has simultaneously allowed for evaluation of the biochemical features. The EC growth ability was assessed at 4°C, 10°C, 45°C in liquid broth (Brain-Heart Infusion, bioMérieux, France) for 24 h. After the incubation time the liquid broth was recultured onto CA and incubated at 37°C in a CO_2_-enriched atmosphere. The growth response was assessed after 24 h and 48 h. The ability to survive at 60°C, 70°C was estimated for 15 min, 30 min, 1 h in liquid broth tubes, followed by incubation (at 37°C, 5%CO_2_) of inoculated CA plates. The results were assessed after 24 h and 48 h.

### Detection of virulence factors

The presence of genes encoding for various virulence factors: *asa1* (aggregation substance), *gelE* (gelatinase), *hyl* (hyaluronidase), *esp* (enterococcal surface protein), *cylA* (cytolisin), *efaA* (endocarditis antigen), *ace* (collagen-binding protein) were determined by PCR in all EC isolates, using primers and conditions previously described [29–31]. The reactions were performed as three duplex PCRs (*asa1/gelE*, *cyl*A*/esp*, *efaA/ace*) and single PCR (*hyl*). For the duplex PCR combinations, one reaction mix (25 μl) contained 12.5 μl DreamTaq PCR Master Mix (Thermo Fisher Scientific Inc., USA), 1.2 μl mix of 4 primers (50 pmol/μl), 4 μl DNA and nucleases-free water. For *hyl* detection, one reaction mix contained 12.5 μl DreamTaq PCR Master Mix (Thermo Fisher Scientific Inc., USA), 0.3 μl of each primer (50 pmol/μl), 4 μl DNA and 7.9 μl nucleases-free water. Amplification conditions were as follows: a first denaturation step of 94°C for 5 min, 30 cycles of denaturation at 94°C for 1 min, annealing at 56°C for 1 min (55°C for *efa*A/*ace*), extension at 72°C for 1 min, followed by an elongation step at 72°C for 10 min. PCR products were separated on 1.2% agarose gels. *Enterococcus faecalis* ATCC 29212 (*asa1*, *gelE*) and *Enterococcus faecalis* ATCC 51299 (*esp*, *cyl*A, *efaA*, *ace*) strains served as positive controls for the virulence factors. Additionally, gelatinase production was detected by inoculating the enterococci onto tubes with Difco Nutrient Gelatin (BD, USA). The tubes inoculated with *S*. *aureus* ATCC 25923 (gelatinase positive), *E*. *coli* ATCC 25922 (gelatinase negative) and an uninoculated tube, as negative control, were used for comparison. The incubation was conducted aerobically at 37°C for 21 days. All tubes were examined for solidification or liquefaction according to the procedure provided by the manufacturer. The results were recorded after 24 h incubation, then at 7, 10, 14, 21 day.

### Molecular typing

The results of the phenotypic identification methods were verified using superoxide dismutase (*sodA*) partial gene sequencing. A 371-bp gene fragment of the *sodA* was amplified using primers and conditions described previously [32]. PCR products were sequenced (IBB PAN, Genomed, Poland). To study diversity of EC within poultry type, the sequences were subjected to phylogenetic analysis. The sequences of CB, BB, CL, T and waterfowl were aligned separately using ClustalW program and phylogenetic trees for each poultry group were constructed using Neighbor joining in the MEGA 7 package [33].

### Pulsed Field Gel Electrophoresis—PFGE

PFGE was done as previously described [26, 34–36]. Genomic DNA from all isolates was embedded in 2% agarose plugs (InCert Agarose, Lonza, Rockland, USA). DNA was digested with the *Sma*I enzyme (20 U/μl; Fermentas, Lithuania). Electrophoresis of digested fragments was carried in 1% agarose on CHEF DRII system (Bio-Rad Laboratories, Berkeley, CA, USA) using 0.5xTBE at 14°C. The initial and final switch time was 0.5 and 35 s respectively, at 6V/min for 24 h. The DNA banding patterns were analysed with Gel Compar II BioNumerics v. 7.0 software (Applied Maths, Belgium) and cluster analysis was performed by UPGMA based on the Dice similarity coefficient, with optimization and position tolerance set at 1%. EC isolates were clustered using > 80% homology cut-off, above which strains were considered to be closely related and assigned to the same PFGE type. The reference strain *E*. *cecorum* ATCC 43198 was used as control.

### Statistical analyses

Numerical variables were presented as a median, inter quartile range (IQR) and range, and compared between groups with a Mann-Whitney U test. Categorical variables were given as counts and percentages, which were then compared between groups using a Pearson chi-square test. As five groups were compared a chi-square test was an omnibus test. Therefore, when it yielded significant result, a post hoc analysis was performed according to the procedure described by Markowski and Markowski [37]. Briefly, the group with the largest average contribution to the chi-square total was identified, then this group was removed from the contingency table and a chi-square test was performed again. The procedure was repeated until a chi-square test yielded an insignificant result. All statistical tests were two-sided. A significance level (α) was set at 0.05. Statistical analysis was performed in Statistica 12.5 (StatSoft Inc.).

## Results

CB were significantly younger at the moment of diagnosis compared to other birds (Table 1).

**Table 1:** The age of birds at which infection was diagnosed.

Type of affected flock	Age (days)		
	Median	IQR	Range
CB	24.0	20.0–32.0	0–49
BB	203.0	77.0–294.0	6–448
CL	189.0	84.0–224.0	21–511
T	63.0	63.0–84.0	56–140
W (G & D)	42.0	23.0–280.0	17–730
G	45.5	28.0–280.0	23–730
D	-	-	17

CB—Commercial Broiler Chickens; BB—Broilers Breeders; CL—Commercial Layers; T—Turkeys; W—Waterfowl; G—Geese; D—Ducks; IQR—Inter quartile range

Disease outbreaks were reported in all but two mountain voivodeships (14/16, 87.5%). All poultry types had locomotor problems, and cachexy depending on the course of diseases. In opposition to other poultry types, clinical nervous signs (tremor, unsteady walk) were observed only in ducks, while decreased reproduction in geese. Diseases resulted in uneven growth, poor uniformity, increased mortality and losses. Moreover, embryonic mortality in CB and deformed eggs in CL were observed. Necropsy revealed the presence of spinal abscesses only in CB and BB flocks. Purulent arthritis and femoral head necrosis (FHN) were noted in CB, BB, arthritis in CL, T, G, D. All groups of poultry showed fibrinous pericarditis, hydropericardium, fibrinous hepatitis, and also fibrinous pneumonia with exception of D. Moreover, T revealed lesions in the infraorbital sinus. Geese showed lesions mainly in the heart and lungs, while ducks showed congested brain and osteomyelitis of long bones. Only BB and CL revealed fibrinous ovaritis, congested oviduct, gassed intestinal contents, watery contents in caeca. Urate deposits in ureters were found in BB and G, while gout in CL. Ascites was observed only in CB and BB. In CB flocks, isolates were more often retrieved from the bone marrow, joints and spine, in BB flocks from the lungs and in CL flocks from the trachea (Table 2).

**Table 2:** The incidence [n (%)] of isolation clinical *E*. *cecorum* from different tissue samples depending on the poultry flocks.

Tissues	CB	BB	CL	T	W	Total	p-value
Heart	29 (38.2)	11 (29.7)	13 (56.5)	3 (60.0)	3 (42.9)	59 (39.9)	0.270
Bone marrow	55 (72.4)↑	7 (18.9)	3 (13.0)	0	2 (28.6)	67 (45.3)	<0.001*
Joints	27 (35.5)↑	1 (2.7)	1 (4.3)	0	2 (28.6)	31 (21.0)	<0.001*
Spine	26 (34.2)↑	3 (8.1)	1 (4.3)	0	0	30 (20.3)	0.001*
Liver	11 (14.5)	3 (8.1)	3 (13.0)	0	3 (42.9)	20 (13.5)	0.186
Spleen	1 (1.3)	0	0	0	0	1 (0.7)	-
Lungs	5 (6.6)	15 (40.5)↑	5 (21.7)	1 (20.0)	1 (14.3)	27 (18.2)	0.001*
Suborbital sinus	0 (0)↓	2 (5.4)	2 (8.7)	1 (20.0)	1 (14.3)	6 (4.0)	0.044*
Trachea	0 (0)	2 (5.4)	4 (17.4)↑	0	1 (14.3)	7 (4.7)	0.008*
Air sacs	0	2 (5.4)	0	0	0	2 (1.3)	0.229
Mixed tissues	11 (14.5)	8 (21.6)	3 (13.0)	0	0	22 (14.9)	0.274
Yolk sac	1 (1.3)	1 (2.7)	0	0	0	2 (1.3)	-
Embryos	2 (2.6)	0	0	0	0	2 (1.3)	-
Ovary	0	0	1 (4.3)	0	0	1 (0.7)	-
Brain	0	0	0	0	1 (14.3)	1 (0.7)	-
Eggs	0	0	1 (4.3)	0	0	1 (0.7)	-
Total (n)	76	37	23	5	7	148	-

CB—Commercial Broiler Chickens; BB—Broilers Breeders; CL—Commercial Layers; T—Turkeys; W—Waterfowl; G—Geese; D—Ducks

*p-value < 0.05 refers to statistically significant.

### Biochemical features, survival and the growth abilities

Results for 32 biochemical parameters were presented in Table 3. Compared to manufacturer’s recommendations, clinical EC showed increased βGAR, βGAL, MLZ, and decreased MAN. *E*. *cecorum* isolated from CB were significantly more often positive for βGAL, βNAG, MLZ, and less often positive for PAL and βMAN than isolates from other poultry types. CB isolates were significantly more frequently LAC positive than isolates from BB, CL. *E*. *cecorum* from T were significantly less frequently positive for βNAG, βGAL and RIB. Among chicken isolates (CB, BB, CB), MAN and GTA tests were significantly more often demonstrated in EC-isolates from BB than CB and CL flocks.

**Table 3:** Positive reactions [n, (%)] in rapid ID 32 STREP exhibited by clinical *E*. *cecorum* of different poultry types.

Parameter	CB	BB	CL	T	W	Total	p-value
ADH (argininedihydrolase)	1	0	0	0	0	1 (0.7)	-
βGLU (β-glucosidase)	76 (100)	37 (100)	23 (100)	5 (100)	7 (100)	148 (100)	-
βGAR (β-galactosidase)	49 (64.5)	17 (46.0)	15 (65.2)	1 (20.0)	3 (42.9)	85 (57.4)	0.105
βGUR (β-glucuronidase)	76 (100)	26 (70.3)↓	19 (82.6)↓	5 (100)	7 (100)	133 (89.9)	0.001*
αGAL (α-galactosidase)	75 (98.7)	37 (100)	23 (100)	5 (100)	7 (100)	147 (99.3)	-
PAL (alkaline phosphatase)	56 (73.7)↓	33 (89.2)	22 (95.7)	5 (100)	7 (100)	122 (82.4)	0.023*
RIB (ribose)	75 (98.7)	37 (100)	23 (100)	2 (40.0)↓	7 (100)	144 (97.3)	0.001*
MAN (mannitol)	2 (2.6)	11 (29.7)↑	2 (8.7)	1 (20.0)	2 (28.6)↑	18 (12.2)	0.024*
SOR (sorbitol)	4 (5.3)	3 (8.1)	2 (8.7)	1 (20.0)	1 (14.3)	11 (7.4)	0.764
LAC (lactose)	70 (92.1)	26 (70.3)	18 (78.3)	4 (80.0)	6 (85.7)	124 (83.8)	0.053
TRE (trehalose)	76 (100)	37 (100)	22 (95.7)	5 (100)	7 (100)	147 (99.3)	-
RAF (rafinose)	76 (100)	37 (100)	23 (100)	5 (100)	7 (100)	148 (100)	-
VP (VogesProskauer, aceton production)	47 (61.8)	23 (62.2)	18 (78.3)	3 (60.0)	6 (85.7)	97 (65.5)	0.414
APPA (alanyl-phenyl alanyl-prolinearylamidase)	0	0	1 (4.4)	0	0	1 (0.7)	-
βGAL (β-galactosidase)	69 (90.8)↑	26 (70.3)	15 (65.2)	1 (20.0)↓	4 (57.1)	115 (77.7)	<0.001*
PYRA (pyroglutamic acidarylamidase)	0	0	0	0	0	0	-
βNAG (N-acetyl-β-glucosaminidase)	71 (93.4)↑	24 (64.9)	15 (65.2)	0 (0)↓	3 (42.9)	113 (76.4)	<0.001*
GTA (glycyl-tryptophanarylamidase)	72 (94.7)	37 (100)	20 (87.0)	4 (80.0)	6 (85.7)	139 (93.9)	0.094
HIP (hydrolysis of hipurate)	0	0	0	0	0	0	-
GLYG (glycogen)	13 (17.1)	3 (8.1)	3 (13.0)	2 (40.0)	2 (28.6)	23 (15.5)	0.334
PUL (pullulane)	1 (1.3)	0	1 (4.4)	0	2 (28.6)	4 (2.7)	-
MAL (maltose)	75 (98.7)	36 (97.3)	22 (95.7)	5 (100)	7 (100)	145 (98.0)	0.868
MEL (melibiose)	75 (98.7)	35 (94.6)	22 (95.7)	5 (100)	7 (100)	144 (97.3)	0.673
MLZ (melezitose)	74 (97.4)↑	24 (64.9)	15 (65.2)	2 (40.0)	5 (71.4)	120 (81.1)	<0.001*
SAC (saccharose)	76 (100)	37 (100)	23 (100)	5 (100)	7 (100)	148 (100)	-
LARA (L-arabinose)	0	0	0	0	0	0	-
DARL (D-arabitol)	0	1(2.7)	0	0	0	1 (0.7)	-
MβDG (methyl-βD-glucopyranoside)	75 (98.7)	36 (97.3)	23 (100)	5 (100)	7 (100)	146 (98.6)	-
TAG (tagatose)	53 (69.7)	22 (59.5)	11 (47.8)	2 (40.0)	4 (57.1)	92 (62.2)	0.283
βMAN (β-mannosidase)	7 (9.2)↓	10 (27.0)	7 (30.4)	2 (40.0)	3 (42.9)	29 (19.6)	0.017*
CDEX (cyclodextrin)	76 (100)	37 (100)	21 (100)	5 (100)	7 (100)	146 (98.6)	-
URE (urease)	3 (4.0)	0	1 (4.4)	0	1 (14.3)	5 (3.4)	-

CB—Commercial Broiler Chickens; BB—Broilers Breeders; CL—Commercial Layers; T—Turkeys; W—Waterfowl; G—Geese; D—Ducks.

*p-value < 0.05 refers to statistically significant.

**Fig 1:**
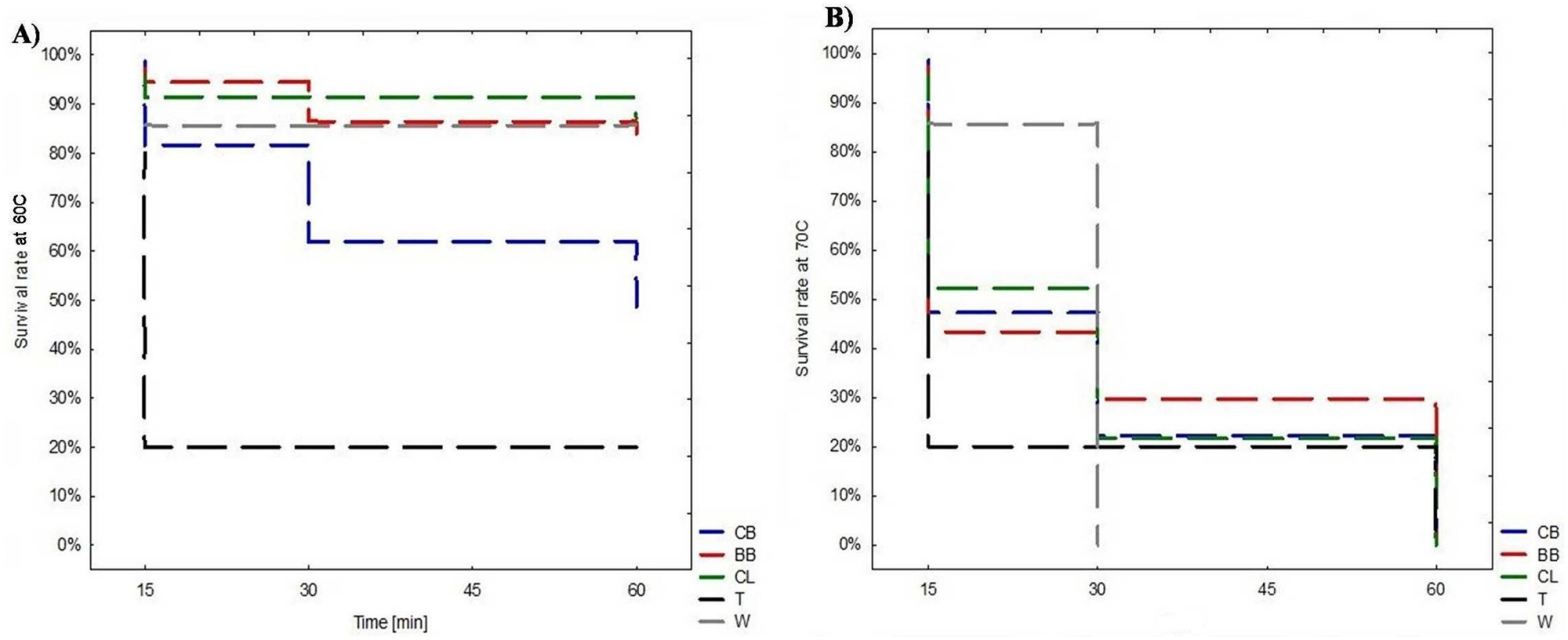
Survival skills of clinical *E*. *cecorum* isolates. (CB—Commercial Broiler Chickens; BB—Broilers Breeders; CL—Commercial Layers; T—Turkeys; W—Waterfowl) after treatment with: A) 60°C for 15min, 30 min, 1 h; and B) 70°C for 15min, 30 min, 1 h.

EC-isolates from CB showed lower ability to survive at 60°C (30min, 1 h) than isolates from BB, CL or other poultry species. EC-isolates from T revealed significantly lower ability to survive at 60°C (15 min, 30 min, 1 h) compared to EC-isolates of other poultry types (Fig 1A). There were no significant differences in the capacity for survival at 70°C and growth at 4°C, 10°C, 45°C between isolates of various poultry groups (Fig 1B, Table 4).

**Table 4:** The ability of clinical *E*. *cecorum* [n, (%)] originated from different poultry to growth at different temperatures.

Type of poultry	Growth at		
	4°C	10°C	45°C
CB (n = 76)	76 (100)	73 (96.1)	50 (65.8)
BB (n = 37)	37 (100)	37 (100)	29 (78.4)
CL (n = 23)	23 (100)	22 (95.7)	19 (82.6)
T (n = 5)	5 (100)	5 (100)	4 (80.0)
W (n = 7)	7 (100)	7 (100)	5 (71.4)
Total (n = 148)	148 (100)	144 (97.3)	107 (72.3)
p-value	-	0.512	0.439

CB—Commercial Broiler Chickens; BB—Broilers Breeders; CL—Commercial Layers; T—Turkeys; W—Waterfowl; G—Geese; D—Ducks

### Detection of virulence factors

Differences in distribution of virulence factors among clinical *E*. *cecorum* of five poultry groups were not statistically significant (Table 5).

**Table 5:** Incidence [n, (%)] of the virulence factors in clinical *E*. *cecorum* depending on the poultry type.

Virulence factor	CB	BB	CL	T	W	p-value
*asa*1 (aggregation substance)	11 (14.5)	3 (8.1)	5 (21.7)	0	0	0.223
*gel*E (gelatinase)	11 (14.5)	3 (8.1)	5 (21.7)	0	0	0.223
*hyl* (hyaluronidase)	0	0	0	0	0	-
*esp* (enterococcal surface protein)	0	0	0	0	0	-
*cyl*A (cytolisin)	0	2 (5.4)	0	0	0	
*efa*A (endocarditis antigen)	6 (7.9)	2 (5.4)	2 (8.7)	0	0	0.723
*ace* (collagen-binding protein)	4 (5.3)	2 (5.4)	2 (8.7)	0	0	0.781
Any virulence factor	12 (8.1)	3 (2.0)	5 (3.4)	0	0	0.198

CB—Commercial Broiler Chickens; BB—Broilers Breeders; CL—Commercial Layers; T—Turkeys; W—Waterfowl; G—Geese; D—Ducks

After exposure to cold temperature, all Difco Nutrient Gelatin tubes inoculated with *E*.*cecorum* exhibited negative reaction for gelatin hydrolysis as shown by solid medium. Gelatin hydrolysis was negative after 24 h and longer incubation. Sequences for representative PCR products (>200 bp) of the virulence genes were deposited in GenBank under the following accession numbers, *asa1*: KY593921, KY613928, KY613929; *gelE*: KY613924, KY613931, KY613932; *cylA*: KY613925; *efaA*:KY613926, KY613930; ace: KY613927.

### Genetic diversity of *E*. *cecorum* of each poultry type based on *sodA* gene sequence

All isolates were confirmed as *E*. *cecorum* by species-specific PCR and sequencing of *sodA* gene fragment. Isolates from each poultry type were analyzed to determine the extent of diversity within a single poultry type. Phylogenetic analysis supported the separation of CB clinical isolates into two genetic lineages: I (comprising 97.4% isolates), and II (2.6% isolates). Five isolates of lineage I (6.8%) belonged to subgroup I’ (S1 Fig). BB isolates were separated into the main lineage I (91.9% isolates), and II, III, IV (each with 2.7% isolates). Nine isolates of lineage I (26.5%) were classified as subgroup I’(S2 Fig). The phylogenetic analysis placed CL isolates into three main lineages: I (comprising 87% isolates), II (4.3%), III (8.7%) (S3 Fig). Isolates from T flocks were distributed into the major lineage I (80%) and II (20%) (S4 Fig). Similarly, W isolates were assigned to two lineages: I (85.7% isolates) and II (14.3%) (S5 Fig).

### Genetic diversity of *E*. *cecorum* of each poultry type based on PFGE profile

**Fig 2:**
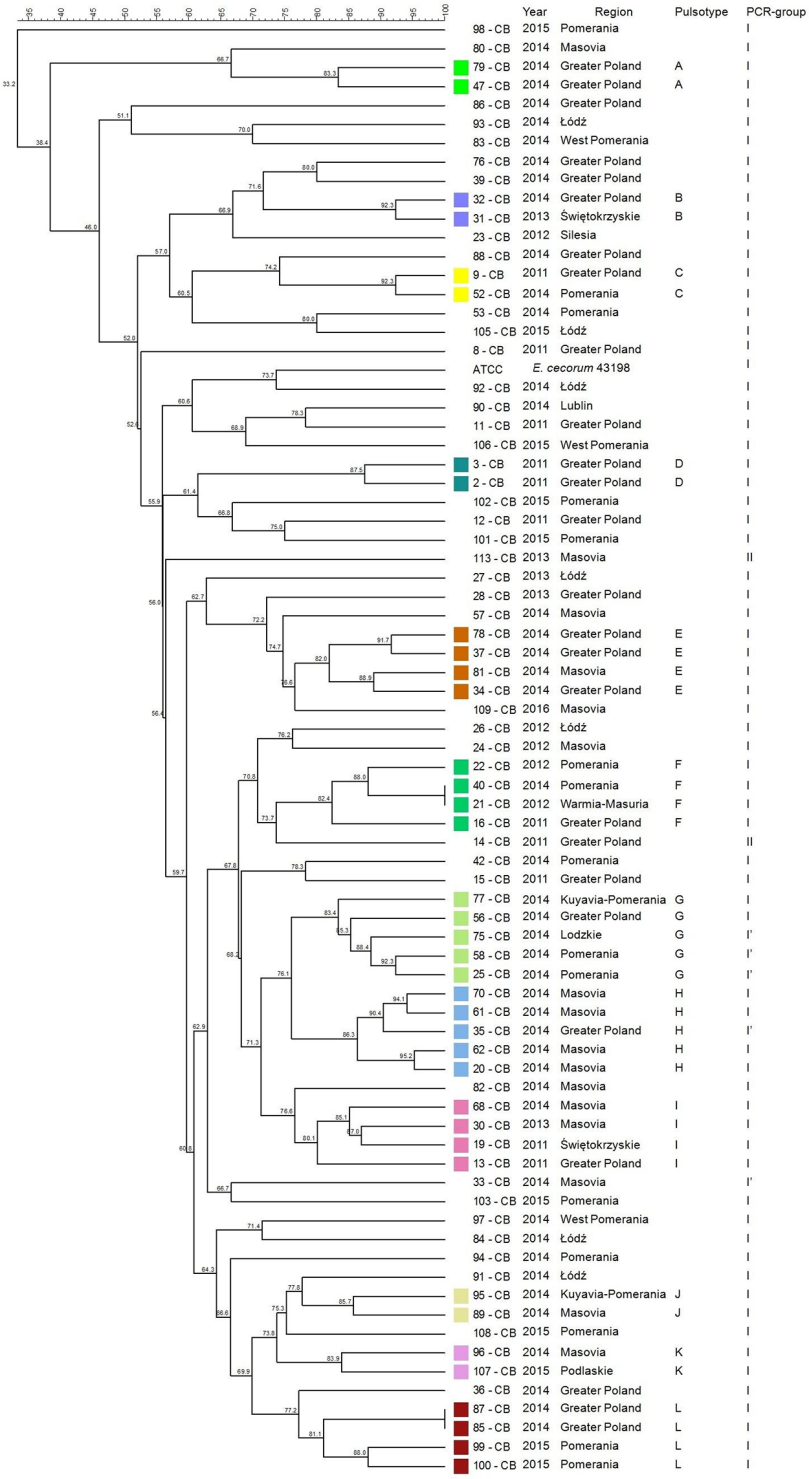
Tree showing the genetic similarity between pathogenic *E*. *cecorum* isolates from 76 broiler chickens flocks (CB) based on PFGE (*Sma*I) and PCR results (sequences of *sodA* gene fragment). The each pulsotype is shown with the corresponding number of isolate, year of isolation, location of affected flock, and PCR-group (*sodA*). Analysis revealed 2 phylogenetic groups (I–II, and I’ subgroup), and 12 (A–L) individual pulsotypes comprised 38 CB isolates. Dendogram was constructed based on the Dice similarity coefficient and the UPGMA clustering method. *Enterococcus cecorum* ATCC 43198 was used as a reference strain.

The genetic diversity analysis based on the dendrogram calculated from the PFGE patterns clustered 38 CB isolates (38/76, 50%) into 12 distinct profiles (A-L). All of them belonged to the group I or subgroup I’ typed by PCR (Fig 2). Some of clustered isolates had the same year of isolation and geographical origin (pulsotype A, D). Other isolates of the same year have been distributed between different geographical regions (G, J). In two pulsotypes (E, H), isolates of the same year had the same geographical origin with the exception of one *E*.*cecorum* isolate which was responsible for the outbreak in a different but non-distant region.

**Fig 3:**
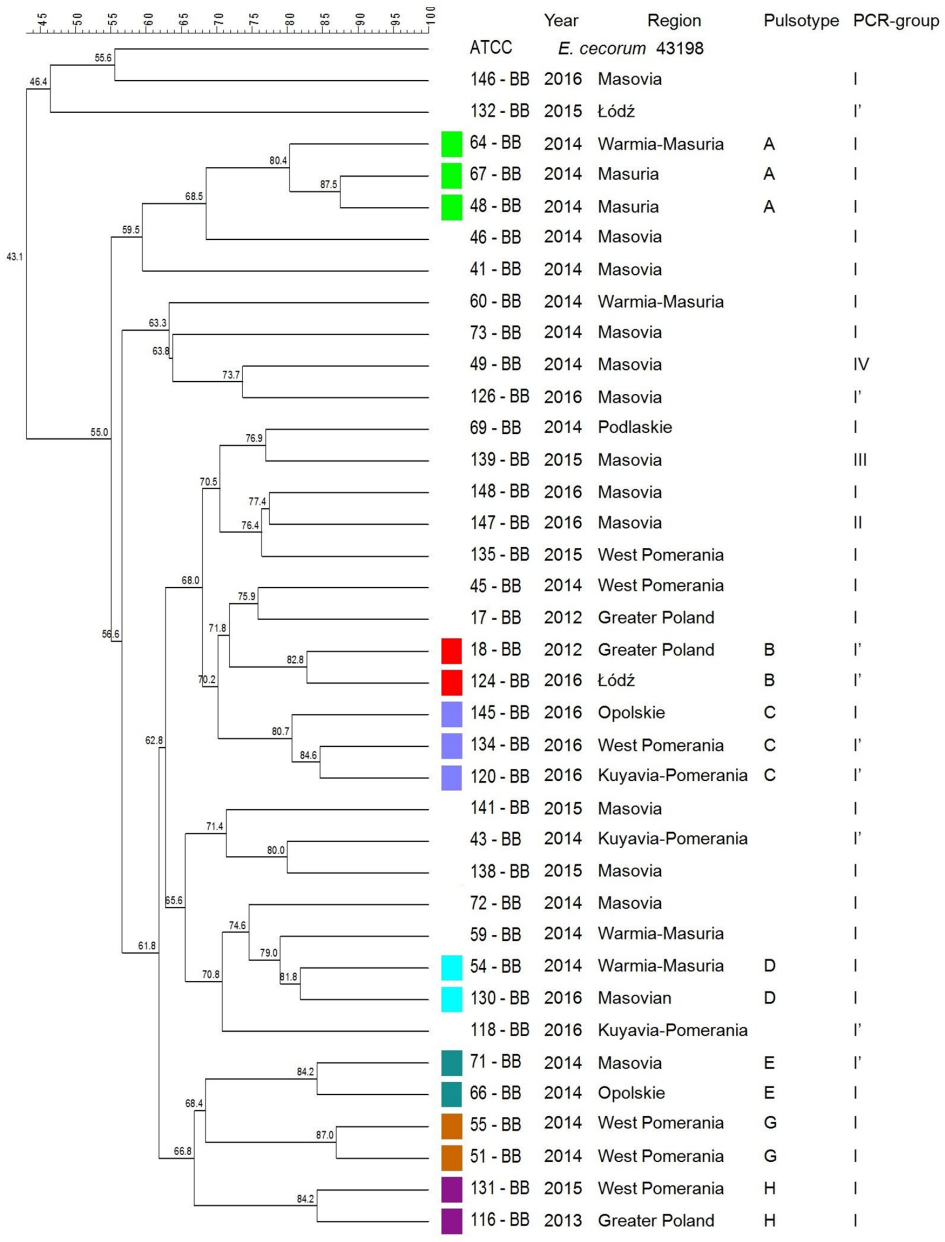
Tree showing the genetic similarity between pathogenic *E*. *cecorum* isolates from 37 broiler breeder flocks (BB) based on PFGE (*Sma*I) and PCR results (sequences of *sodA* gene fragment). Analysis revealed 7 (A–G) individual pulsotypes comprised 37 BB isolates and 4 phylogenetic groups (I–IV, and I’ subgroup). The each pulsotype is shown with the corresponding PCR-group (*sodA*), number of isolate, year of isolation, location of affected flock. Dendogram was constructed based on the Dice similarity coefficient and the UPGMA clustering method. *Enterococcus cecorum* ATCC 43198 was used as a reference strain.

A total of 16 (16/37; 43.2%) clinical EC isolates from BB were clustered into 7 profiles (A-G). Isolates belonged to PCR-group I or subgroup I’ (Fig 3). In 3 pulsotypes (A, C, E), isolates were obtained in the same year (respectively to each pulsotype) but from different geographical regions. More temporal than geographical clustering was observed among BB isolates.

**Fig 4:**
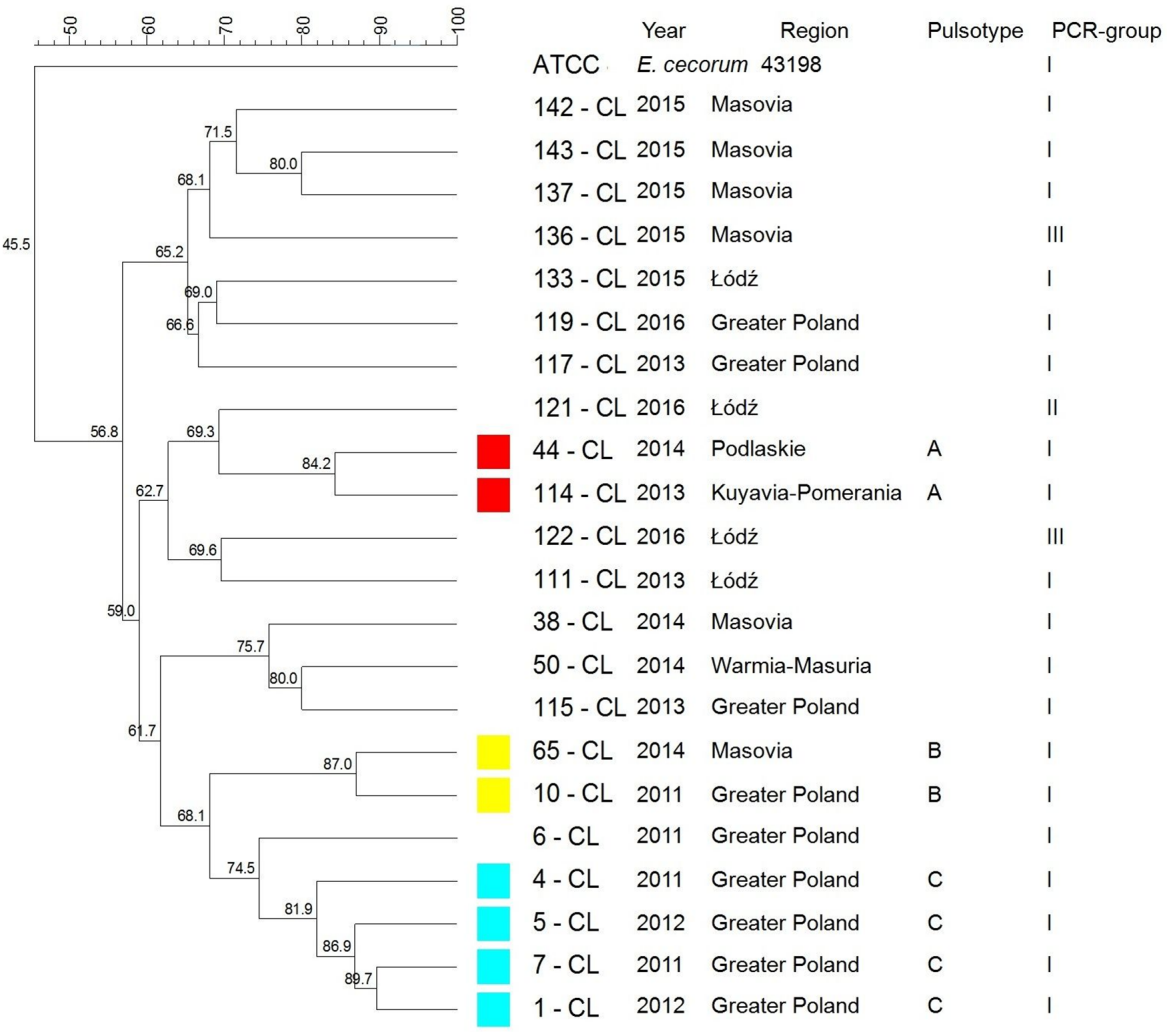
Tree showing the genetic similarity between pathogenic *E*. *cecorum* isolates from 23 layers flocks (CL) based on PFGE (*Sma*I) and PCR results (sequences of *sodA* gene fragment). The each pulsotype is shown with the corresponding PCR-group (*sodA*), number of isolate, year of isolation, location of affected flock. Analysis revealed 3 (A–C) individual pulsotypes comprised 8 CL isolates and 3 phylogenetic groups (I–III). Dendogram was constructed based on the Dice similarity coefficient and the UPGMA clustering method. *Enterococcus cecorum* ATCC 43198 was used as a reference strain.

The PFGE analysis of CL clinical EC yielded 3 pulsotypes (A-C) containing 34.8% (8/23) isolates. All isolates belonged to PCR-group I. Four isolates of the same geographical origin were clustered together (C) (Fig 4).

**Fig 5:**
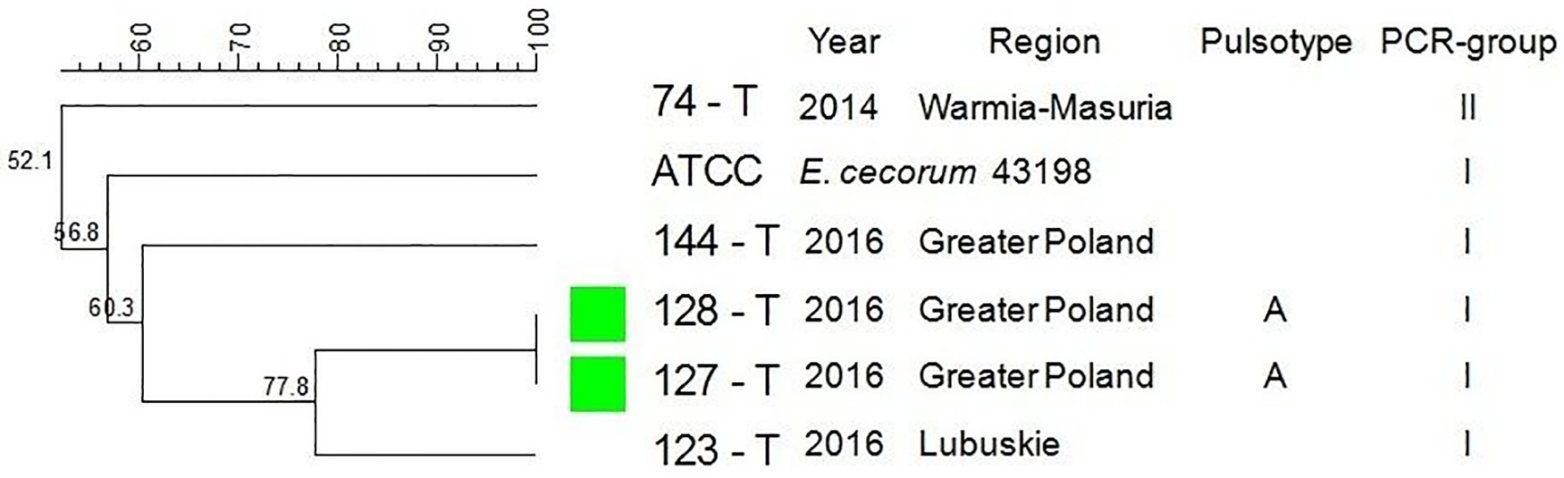
Tree showing the genetic similarity between pathogenic *E*. *cecorum* isolates from 5 turkey flocks (T) based on PFGE (*Sma*I) and PCR results (sequences of *sodA* gene fragment). The each pulsotype is shown with the corresponding PCR-group (*sodA*), number of isolate, year of isolation, location of affected flock. Analysis revealed 1 (A) individual pulsotypes comprised 2 T isolates and 2 phylogenetic groups (I–II). Dendogram was constructed based on the Dice similarity coefficient and the UPGMA clustering method. *Enterococcus cecorum* ATCC 43198 was used as a reference strain.

**Fig 6:**
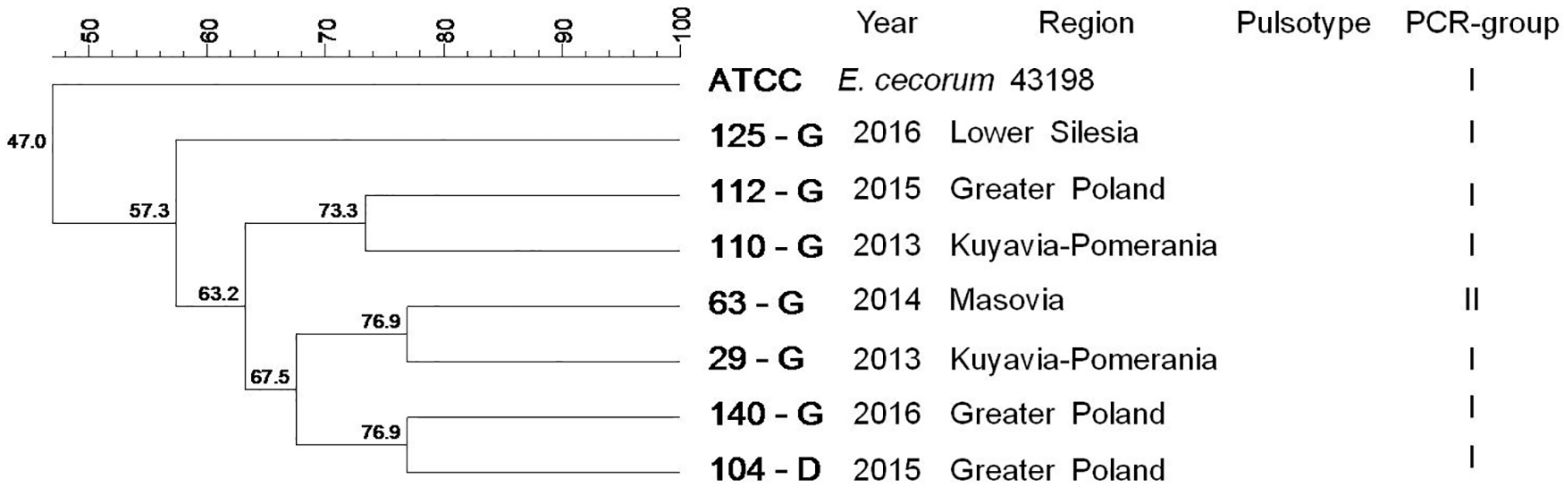
Tree showing the genetic similarity between pathogenic *E*. *cecorum* isolates from 7 waterfowl flocks (W) based on PFGE (*Sma*I) and PCR results (sequences of *sodA* gene fragment). No pulsotypes were found. Phylogenetic analysis showed 2 groups (I-II). The each isolate is shown with the corresponding PCR-group (*sodA*), number, year of isolation, location of affected flock. Dendogram was constructed based on the Dice similarity coefficient and the UPGMA clustering method. *Enterococcus cecorum* ATCC 43198 was used as a reference strain.

Among isolates representing T flocks, two isolates (40%) were clustered in pulsotype A. Isolates which represented the same PCR-group (I) were sampled in the same year and originated from the same voivodeship (Fig 5). PFGE did not distinguish pulsotypes among isolates from waterfowl, and strains were not clustered (Fig 6).

Phylogenetic anaylsis based on both PCR and PFGE revealed the presence of distinct isolates in each poultry type: 2 isolates in CB; 3 isolates in BB and CL; 1 isolate in T and W. These isolates formed separate groups and did not closely cluster with other strains.

## Discussion

*Enterococcus cecorum–*associated infections were reported in several species of poultry with different production purposes [25]. However, there is still little knowledge on the differences between clinical isolates obtained from affected poultry. Based on 2011–2016 field data from Poland we found that *E*. *cecorum* affected commercial poultry kept in intensive production systems, interestingly often on farms with high biosecurity standards (personal communications with poultry veterinarians). EC-associated disease outbreaks were reported in almost all voivodeships (87.5%) of high, as well as low density poultry productions, which may indicate a wide geographic distribution of pathogenic isolates. EC strains were the most prevalent cause of disease among commercial chicken flocks: broiler chickens 51.4% > broiler breeder chickens 25% > layers 15.5%, then waterfowl 4.7%, and turkeys 3.4%. The results would confirm that meat chickens are particularly prone to infection compared to other poultry types. Our study provides the age differences between poultry types at diagnosis of disease, which could indicate differences in age-related predisposition to EC-infection. According to the literature, typical clinical signs of EC infection in broilers and broiler breeders were seen between 5 to10 weeks of age with a marked increase in flock mortality [14, 15, 17, 19]. Similarly to the previous observations [9] infections in CB were diagnosed earlier (3–4 weeks) in this study, however 4% were found during the first week of the growing period (in 1–7 day-old chicks). In our study, infections in BB were found later (29 weeks) than in the literature [13, 14, 21, 22]. On the other hand more than 65% of them were diagnosed in birds > 24 weeks of age. As far as we know this is the first study that describes EC-associated infection in chicken layers and the first in Europe that shows infection in turkeys. In the available literature there is no information about the age or lesions in affected layers. In our study, a high prevalence of infections (62%) was diagnosed in layers >18 weeks of age, mainly at 27 weeks of age. Similarly to other authors we suggested correlation between the age of affected birds with a period of rapid skeletal development [21]. According to the recent literature, the degree of osteochondrosis lesions of the FTV has impact on the appearance of ES lesions in broiler chickens [26]. Based on our observations, *E*. *cecorum* may cause arthritis in all poultry types, however spinal lesions were only found in CB, BB. Isolates were significantly more frequently retrieved from joints and spine in CB than from respiratory system when compared to other poultry groups. The predisposition of meat chickens to enterococcal vertebral osteomyelitis may be related to genetic factors (selection for rapid growth) and anatomical features in the FTV. The increased calcium requirements during the pre-laying period and the onset of laying period could have impact on disclosure of clinical form of infection during laying period. Further studies are needed to identify risk factors for EC-disease outbreak in different poultry types. Ducks were prone to early infection and–not typical for *E*. *cecorum–*lesions in central nervous system. Our field observations were confirmed by previous experimental study by Jung et al. [24]. Some differences in clinical and post-mortem manifestation of infection in different poultry and thus differences in pathogenesis seem to exist. Although transovarian transmission was not examined in this study, single pathogenic *E*. *cecorum* strains were derived from dead-in shell-embryos, ovary and deformed eggs. So far, vertical transmission of pathogenic strains has not been definitively demonstrated [15, 20, 38].

Our study showed differences between clinical EC of various poultry types with respect to 10 biochemical parameters (out of total 32): βGUR, PAL, RIB, MAN, βGAL, βNAG, MLZ, βMAN, LAC, GTA. *E*. *cecorum* spinal isolates were found to be incapable of mannitol metabolism compared with commensal strains, which was additionally confirmed by comparative genomic analysis [27, 39]. Other authors demonstrated a lack of mannitol utilization in pathogenic isolates originated from broilers, broiler parent chicks, turkeys and ducks [20, 25]. Our results were in agreement with the published findings, however we showed significant differences in mannitol metabolism between isolates from various poultry groups. BB and W isolates exhibited significantly higher capacity for mannitol metabolism compared to CB, CL and T. It seems that mannitol does not serve as a source of energy (major carbon source) for pathogenic *E*. *cecorum*. The importance of inability of clinical *E*. *cecorum* for mannitol fermentation is not known.

In opposition to other authors, we showed the resistance of all clinical *E*. *cecorum* to low temperatures [1, 3, 40] and relatively high ability to grow at 45°C. The study revealed significant differences in the survival at high temperature (60°C) between EC of various poultry groups. It seems that CB and T isolates have lower ability to survive at 60°C. We confirmed high sensitivity of all isolates to prolonged exposure to 70°C, which is consistent with previous observations [28].

Recent study demonstrated the presence of very few virulence factors in both pathogenic and commensal poultry EC-isolates [25, 41], however pathogenic strains seemed to be more virulent than commensal [42]. In our study only chicken EC isolates harbored virulence genes, however the prevalence of virulence factors was relatively low. The highest prevalence of these genes among *E*. *cecorum* isolates was found in CB (8.1%) > BB (3.4%) > CL (2%). Our findings confirmed the presence of a*sa1*, *gelE*, *efaA*, *ace* genes in strains obtained from CB, BB, CL. Only BB isolates harbored *cylA*. It seems that virulence factors are not required to determine pathogenicity of the EC-isolate. Similarly to previous observations [28, 41], we did not detect *hyl* gene, however Jung et al. [25] described *hyl* in 14.3% of pathogenic isolates collected from broiler and ducks.

Although phylogenetic analysis by *sod*A sequencing showed weak differentiation between isolates, it revealed a few unique, non-clustered isolates which formed separate groups.

Previous studies have revealed the existence of genetic polymorphism among EC associated with clinical disease [28, 36, 39, 41]. According to other authors, genotypes of clinical isolates have been found to be more similar to each other than to non-clinical isolates [15, 35, 38, 39]. In the present study, PFGE indicated at the genetic heterogeneity among pathogenic EC involved in outbreaks in each poultry type. A total of 12 CB, 7 BB, 3 CL and 1 T clusters representing respectively 38 of EC-disease outbreaks in CB, 16 outbreaks in BB, 8 in CL, 2 in T were detected. We indicated at the possibility that EC outbreaks among CB flocks between 2011–2016 could not have been caused by the same clone, but by several various clones. Similar observations were made for outbreaks among BB, CL, and T flocks. It seems that EC outbreaks in waterfowl were caused by unrelated isolates which did not form PFGE clusters. Kense et al. [38] reveled that *E*. *cecorum* populations at the broiler farm level showed higher genetic diversity compared with the breeder farm level. In our study, broiler isolates showed greater genetic variability, than isolates from other poultry groups. We confirmed that, within one year in one region either one or several (up to 6) various genetic types of EC may have been involved in outbreaks in CB flocks. In comparison, outbreaks in BB, CL flocks were caused by one or two genetic types of *E*. *cecorum* and in T flocks by a single genetic type of *E*. *cecorum*. Moreover, outbreaks among CB, BB reported in following years in the same region, were usually caused by distinct set of genetic types of *E*. *cecorum* e.g.in CB: Greater Poland in 2011, pulsotypes C, D, F, I, then in 2014: A, B, E, G, H, L; Pomerania Province in 2012, pulsotypes: F, 2014: C, F, G, but in 2015 pulsotype L; in BB: Greater Poland, 2012, pulsotype B, but in 2013: H; West Pomeranian in 2014 pulsotype G, in 2015: H. On the other hand, some CB (or BB) isolates of the same year and genetic type, may be closely related with EC responsible for outbreaks in several different geographical regions.

To our best knowledge, this is the first study which provides a comparison between the pathogenic *E*. *cecorum* originating from five separate poultry groups. The study highlights the problem of EC infection in different poultry types, and provides a new insight into *E*. *cecorum* as pathogen of different bird species. Previously a separate evolution of pathogenic EC from commensal strains has been suggested [25]. In conclusion, our findings indicate that a population of poultry pathogenic EC is not clearly homogeneous. Although most characteristics are similar, we showed some significant differences between EC responsible for diseases in various poultry species with different production purposes. Furthermore, it seems that there are differences among pathogenic EC within each poultry type and between poultry types. The obtained data can be applied to determine the factors influencing the pathogenic capacity of *E*. *cecorum* to cause disease in different specific poultry. Moreover, the results may be useful in further investigation of EC epidemiology and pathogenesis more focused on a specific type of poultry.

## Supporting information

S1 Fig Phylogenetic tree based on partial *sodA* gene sequence analysis, showing the relationships among 76 pathogenic *E*. *cecorum* from broiler chicken flocks (CB). *E*. *cecorum* ATCC 43198 was used as a reference strain. The phylogenetic analysis of *sodA* showed that isolates formed two genetic lineages (I-II) and one subgroup (I’). The evolutionary history was inferred using the Neighbor-Joining method. The percentage of replicate trees in which the associated taxa clustered together in the bootstrap test (1000 replicates) are shown next to the branches. The tree is drawn to scale, with branch lengths in the same units as those of the evolutionary distances used to infer the phylogenetic tree. The evolutionary distances were computed using the Maximum Composite Likelihood method and are in the units of the number of base substitutions per site. Evolutionary analyses were conducted in MEGA7 [33].
(TIF)

S2 Fig Phylogenetic tree for 37 pathogenic *E*. *cecorum* from broiler breeder flocks (BB) constructed using the Neighbor-Joining method to evaluate the distance between partial *sodA* gene sequences of isolates. *E*. *cecorum* ATCC 43198 was used as a reference strain. Analysis showed four genetic lineages (I-IV) and one subgroup (I’). The percentage of replicate trees in which the associated taxa clustered together in the bootstrap test (1000 replicates) are shown next to the branches. The tree is drawn to scale, with branch lengths in the same units as those of the evolutionary distances used to infer the phylogenetic tree. The evolutionary distances were computed using the Maximum Composite Likelihood method and are in the units of the number of base substitutions per site. Evolutionary analyses were conducted in MEGA7 [33].
(TIF)

S3 Fig Neighbor-joining tree constructed from partial *sodA* gene sequences for 23 pathogenic *E*. *cecorum* isolates from commercial layers (CL) flocks and reference strain *(E*. *cecorum* ATCC 43198). Phylogenetic analysis revealed three lineages (I-III). The percentage of replicate trees in which the associated taxa clustered together in the bootstrap test (1000 replicates) are shown next to the branches. The tree is drawn to scale, with branch lengths in the same units as those of the evolutionary distances used to infer the phylogenetic tree. The evolutionary distances were computed using the Maximum Composite Likelihood method and are in the units of the number of base substitutions per site. Evolutionary analyses were conducted in MEGA7 [33].
(TIF)

S4 Fig Phylogenetic tree for 5 pathogenic *E*. *cecorum* from turkey flocks (T) constructed using the Neighbor-Joining method to evaluate the distance between partial *sodA* gene sequences of pathogenic *E*. *cecorum*. *E*. *cecorum* ATCC 43198 was used as a reference strain. Analysis showed that isolates formed two genetic lineages (I-II). The percentage of replicate trees in which the associated taxa clustered together in the bootstrap test (1000 replicates) are shown next to the branches. The tree is drawn to scale, with branch lengths in the same units as those of the evolutionary distances used to infer the phylogenetic tree. The evolutionary distances were computed using the Maximum Composite Likelihood method and are in the units of the number of base substitutions per site. Evolutionary analyses were conducted in MEGA7 [33].
(TIF)

S5 Fig Phylogenetic tree based on partial *sodA* gene sequence analysis, showing the relationships among 7 pathogenic *E*. *cecorum* from waterfowl flocks (W). *E*. *cecorum* ATCC 43198 was used as a reference strain. The phylogenetic analysis of *sodA* showed that isolates formed two genetic lineages (I-II). The evolutionary history was inferred using the Neighbor-Joining method. The percentage of replicate trees in which the associated taxa clustered together in the bootstrap test (1000 replicates) are shown next to the branches. The tree is drawn to scale, with branch lengths in the same units as those of the evolutionary distances used to infer the phylogenetic tree. The evolutionary distances were computed using the Maximum Composite Likelihood method and are in the units of the number of base substitutions per site. Evolutionary analyses were conducted in MEGA7 [33].
(TIF)
