# Supplementary figures and images for: Characterization of pathogenic Enterococcus cecorum from different poultry groups: Broiler chickens, layers, turkeys, and waterfowl
Source: PLoS One. 2017 Sep 21;12(9):e0185199. doi: 10.1371/journal.pone.0185199 (PMC5608366; doi:10.1371/journal.pone.0185199)

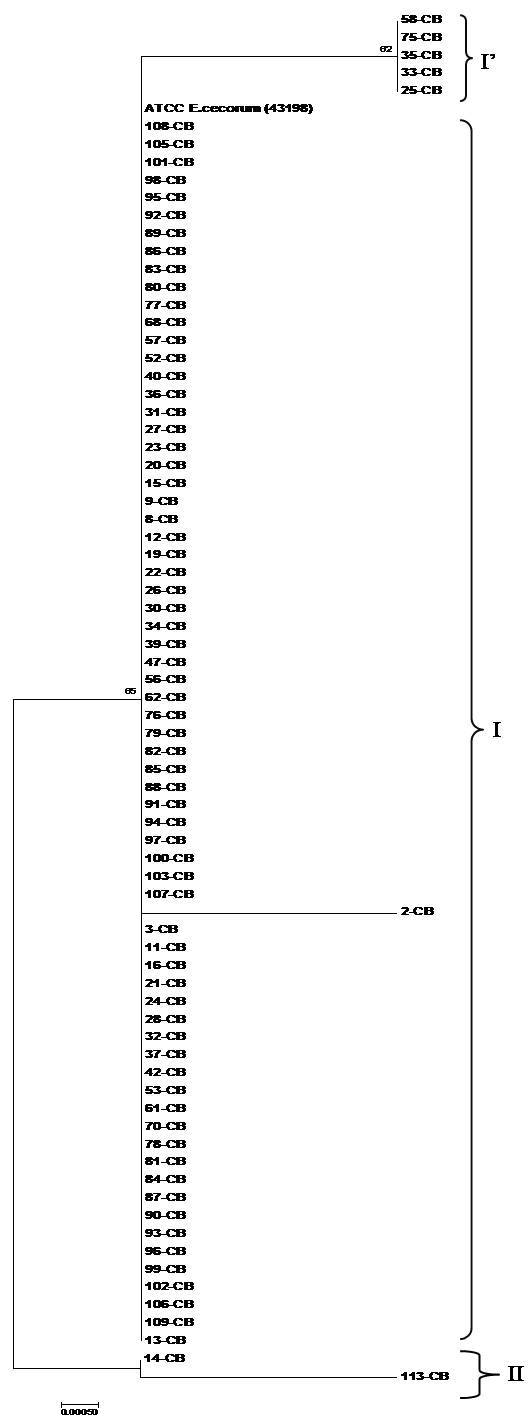

Supplement: S1 Fig — E. cecorum ATCC 43198 was used as a reference strain. The phylogenetic analysis of sodA showed that isolates formed two genetic lineages (I-II) and one subgroup (I’). The evolutionary history was inferred using the Neighbor-Joining method. The percentage of replicate trees in which the associated taxa clustered together in the bootstrap test (1000 replicates) are shown next to the branches. The tree is drawn to scale, with branch lengths in the same units as those of the evolutionary distances used to infer the phylogenetic tree. The evolutionary distances were computed using the Maximum Composite Likelihood method and are in the units of the number of base substitutions per site. Evolutionary analyses were conducted in MEGA7 [33]. (TIF) [file pone.0185199.s001.tif]

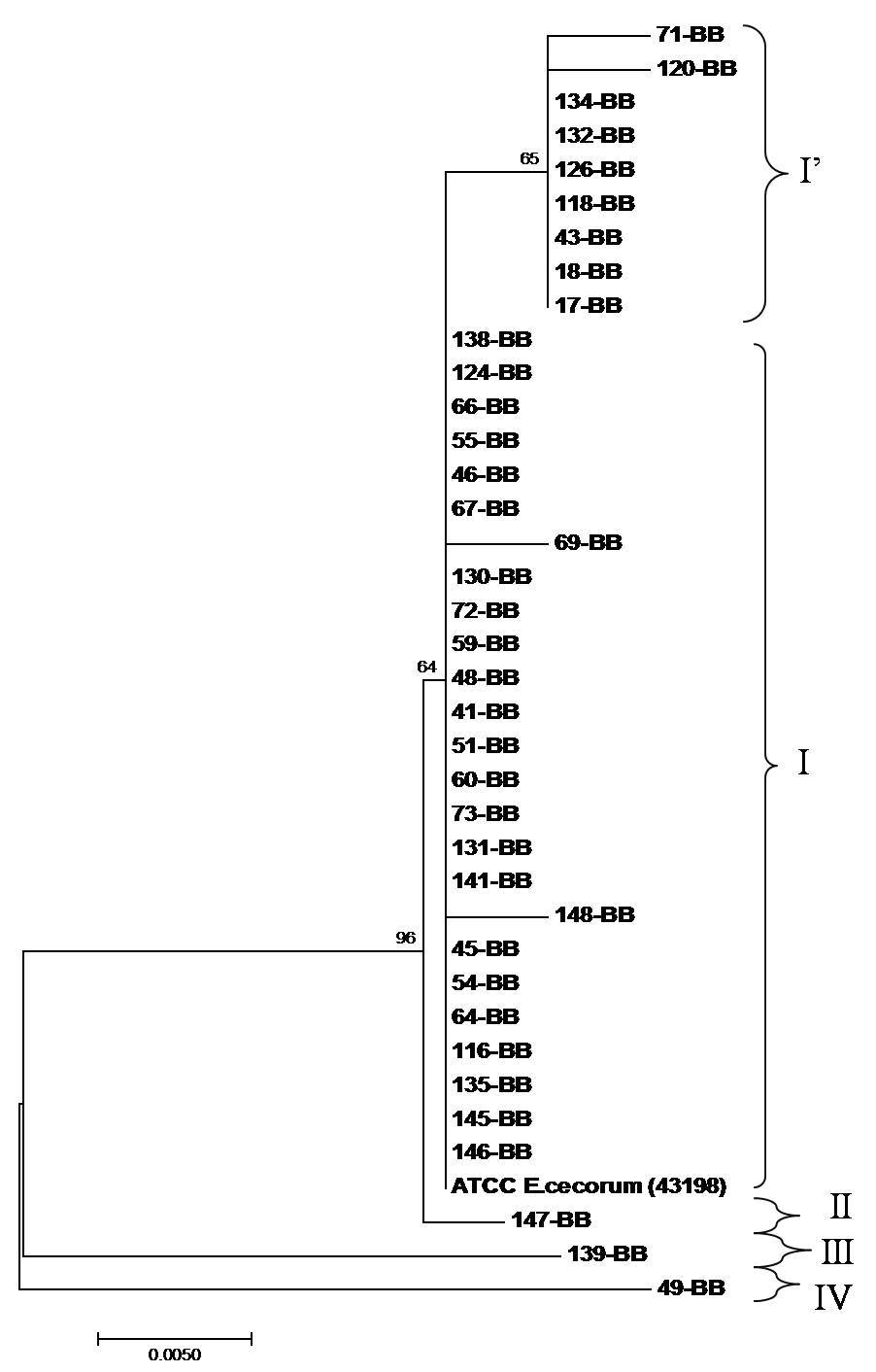

Supplement: S2 Fig — E. cecorum ATCC 43198 was used as a reference strain. Analysis showed four genetic lineages (I-IV) and one subgroup (I’). The percentage of replicate trees in which the associated taxa clustered together in the bootstrap test (1000 replicates) are shown next to the branches. The tree is drawn to scale, with branch lengths in the same units as those of the evolutionary distances used to infer the phylogenetic tree. The evolutionary distances were computed using the Maximum Composite Likelihood method and are in the units of the number of base substitutions per site. Evolutionary analyses were conducted in MEGA7 [33]. (TIF) [file pone.0185199.s002.tif]

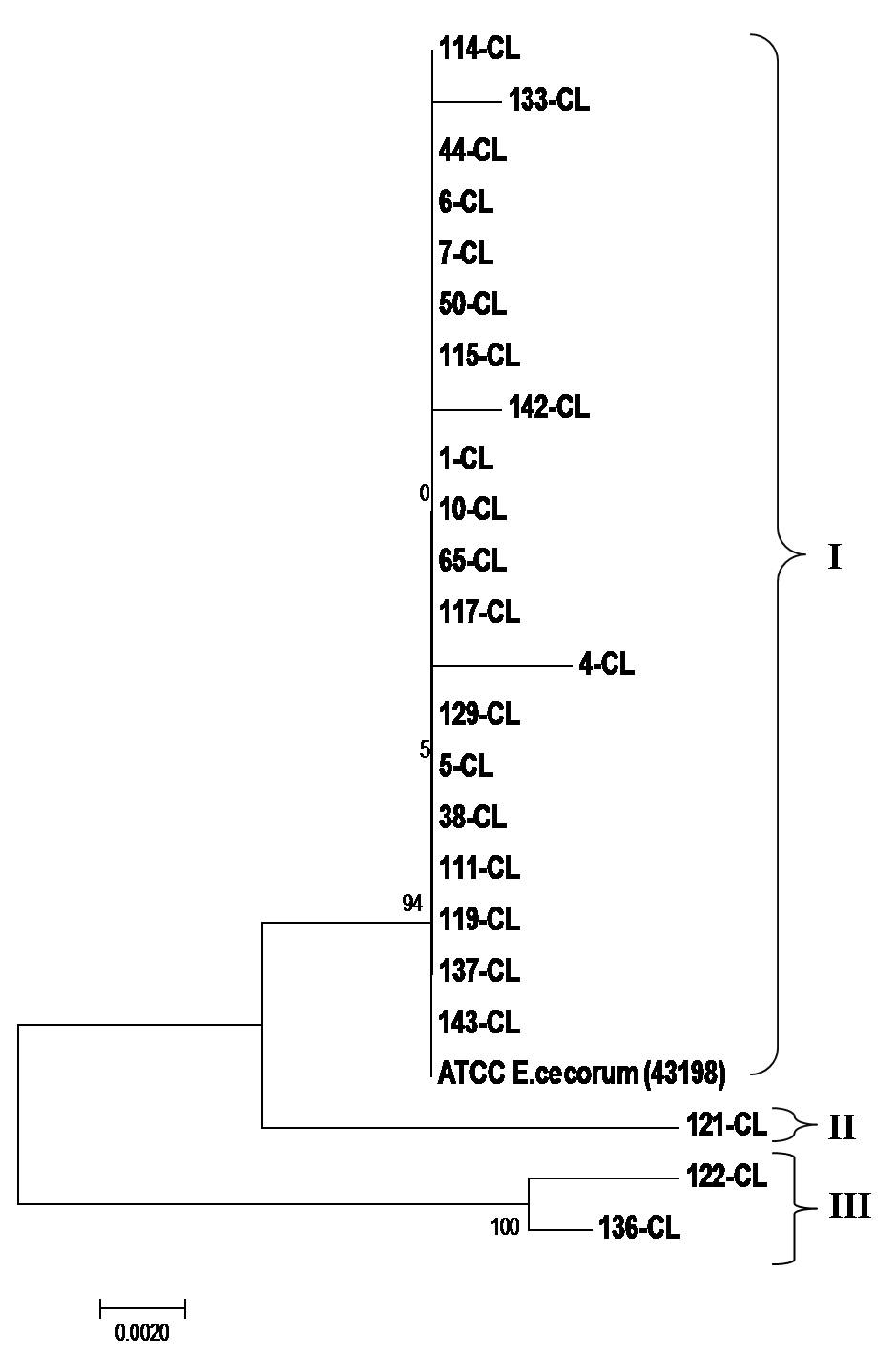

Supplement: S3 Fig — Phylogenetic analysis revealed three lineages (I-III). The percentage of replicate trees in which the associated taxa clustered together in the bootstrap test (1000 replicates) are shown next to the branches. The tree is drawn to scale, with branch lengths in the same units as those of the evolutionary distances used to infer the phylogenetic tree. The evolutionary distances were computed using the Maximum Composite Likelihood method and are in the units of the number of base substitutions per site. Evolutionary analyses were conducted in MEGA7 [33]. (TIF) [file pone.0185199.s003.tif]

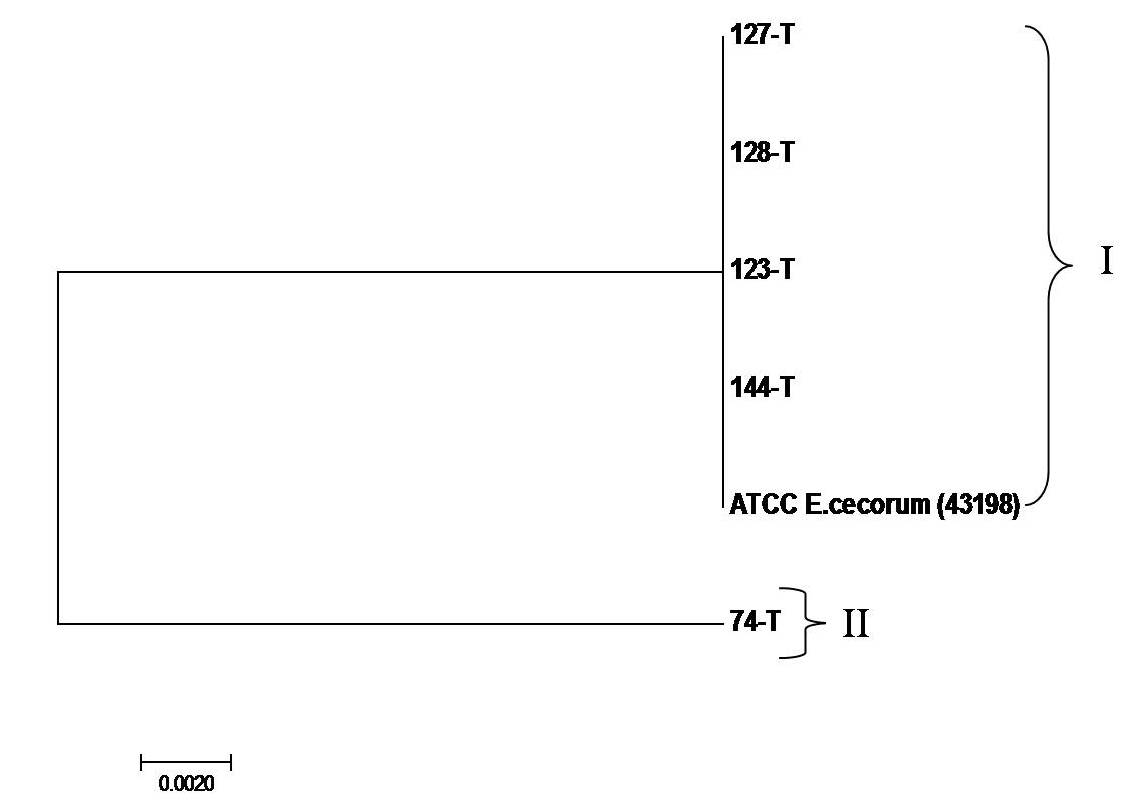

Supplement: S4 Fig — E. cecorum ATCC 43198 was used as a reference strain. Analysis showed that isolates formed two genetic lineages (I-II). The percentage of replicate trees in which the associated taxa clustered together in the bootstrap test (1000 replicates) are shown next to the branches. The tree is drawn to scale, with branch lengths in the same units as those of the evolutionary distances used to infer the phylogenetic tree. The evolutionary distances were computed using the Maximum Composite Likelihood method and are in the units of the number of base substitutions per site. Evolutionary analyses were conducted in MEGA7 [33]. (TIF) [file pone.0185199.s004.tif]

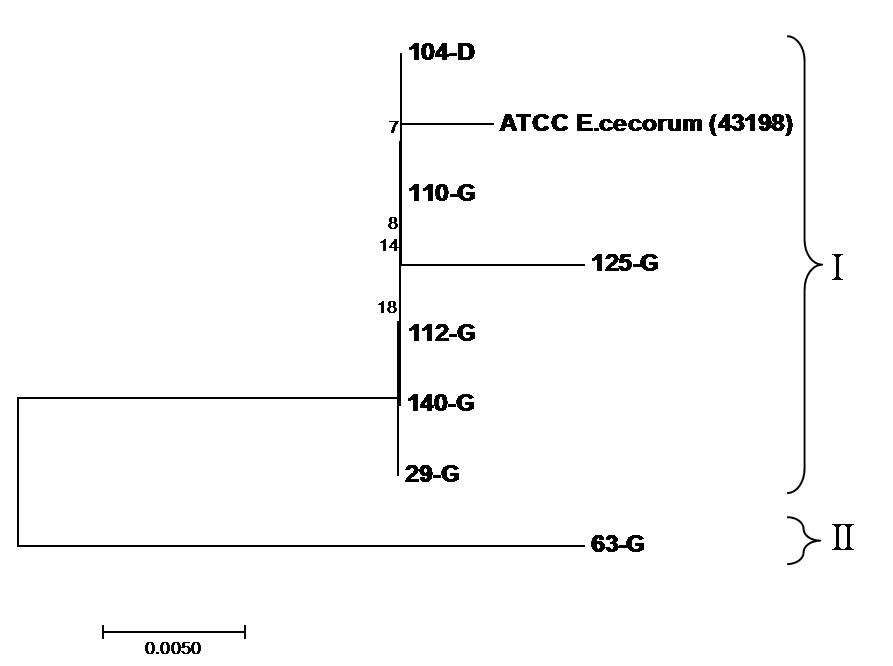

Supplement: S5 Fig — E. cecorum ATCC 43198 was used as a reference strain. The phylogenetic analysis of sodA showed that isolates formed two genetic lineages (I-II). The evolutionary history was inferred using the Neighbor-Joining method. The percentage of replicate trees in which the associated taxa clustered together in the bootstrap test (1000 replicates) are shown next to the branches. The tree is drawn to scale, with branch lengths in the same units as those of the evolutionary distances used to infer the phylogenetic tree. The evolutionary distances were computed using the Maximum Composite Likelihood method and are in the units of the number of base substitutions per site. Evolutionary analyses were conducted in MEGA7 [33]. (TIF) [file pone.0185199.s005.tif]
